# Epidemiology, Clinical Features, and Outcome of Liver Abscess: A Single-Center Experience

**DOI:** 10.7759/cureus.29812

**Published:** 2022-10-01

**Authors:** Vinod Sahu, Dharmendra K Pipal, Yatindra Singh, Vijay Verma, Manisha Singaria, Vibha Rani Pipal, Seema Yadav, Saurabh Jain, Anupam Bhargava

**Affiliations:** 1 General Surgery, Dr. Sampurnanand Medical College, Jodhpur, IND; 2 General, Colorectal, and Minimal-Access Surgery, All India Institute of Medical Sciences Gorakhpur, Gorakhpur, IND; 3 Obstetrics and Gynaecology, All India Institute of Medical Sciences Gorakhpur, Gorakhpur, IND; 4 Anaesthesia, Jaipur National University Medical College, Jaipur, IND; 5 Urology, Sawai Man Singh Medical College, Jaipur, IND; 6 Genitourinary Surgery, Sri Venkateswara Institute of Medical Sciences, Tirupati, IND

**Keywords:** pigtail catheter, klebseilla, pyogenic abscess, amoebic abscess, liver abscess

## Abstract

Introduction

Liver abscesses are rare, but whenever they occur, it is predominantly among males over 60 years of age. The paradigm in the treatment has changed, and percutaneous drainage is now the initial treatment for drainage of the abscesses. Open surgery is reserved for patients with septated abscesses and those greater than 5 cm.

Objective

To study the etiological, clinical, pathological, and demographic characteristics of individuals with liver abscesses and to evaluate the outcome associated with different treatment strategies.

Methods

This clinico-epidemiological study was carried out at a tertiary care hospital in Jodhpur. One hundred patients with liver abscesses were studied. Patients were assigned to three groups: Group 1 - medical management alone (in non-aspirable uncomplicated abscess), Group 2 - USG-guided needle aspiration or pigtail percutaneous catheter drainage plus medical management (in unruptured aspirable abscess), Group 3 - open surgical drainage plus medical management (In ruptured abscesses). Of the total patients, 36% were treated with medical therapy alone, 45% with USG-guided needle aspiration, 10% with USG-guided percutaneous catheter drainage, and 9% with open surgical drainage.

Results

In our study, fever and hepatomegaly were the commonest presentations, observed in 91% and 62% of cases, respectively. *Escherichia coli* *(E.coli*) was the predominant organism cultured in 28 (43.75%) patients followed by *Klebsiella* growing in 24 (37.50%) patients. The right lobe was affected more (83%) than the left lobe and in the majority (83%), a solitary abscess was present. The mean age of liver abscess presentation was 40.72 years, with a 5.67:1 male-to-female ratio. Alcohol consumption was reported by 33% of patients, the majority of whom were men. Serum bilirubin was elevated in 56% of liver abscess patients, while it was normal in 44%. The mean serum bilirubin was 2.08 mg/dl. The mean value in group 1, group 2, and group 3 was 1.44 mg/dl, 2.23 mg/dl, and 2.57 mg/dl, respectively. Liver abscesses were identified in 76% of patients with right lobes; 83% had solitary liver abscesses and 17% had numerous abscesses. Abscess culture showed *E. coli* in 21 (32.81%) and *Klebsiella* in 17 (26.56%) patients.

Conclusion

Right-sided solitary pyogenic liver abscess caused by *E.coli* is the most common liver abscess, with fever and hepatomegaly as the most common presentation. Non-aspirable liver abscesses, regardless of aetiology, can be successfully treated by medical therapy alone. Needle aspiration or catheter drainage is standard for liver abscesses. Thus, needle aspiration has replaced the surgical exploration of liver abscesses.

## Introduction

Liver abscess (LA), which was first described by Hippocrates in 400 BC but first published by Ochsner and colleagues as a review of 47 cases in 1938, is a hepatic parenchymal occupying lesion containing purulent material [1,2]. Various types of abscesses are present in the liver including amoebic, pyogenic, and rarely fungal. In developed countries, pyogenic LA (PLA) constitutes three-fifths, while in developing countries, amoebic LA constitutes two-thirds of total LA cases [3].

LAs can affect anyone at any age, but men between 20 and 40 years of age are most commonly affected by LA. In 60% of cases, the right lobe of the liver is affected. This is because its main blood supply comes from the superior mesenteric vein, which flows straight through the liver [3].

Traditionally, intraabdominal infections such as appendicitis were thought to be the leading cause of LA, but recently, the biliary tree has emerged as the leading risk factor, accounting for 40% of all cases. A PLA is a life-threatening infection with an incidence ranging from 8-22 per 1,000,000 people depending on different geographical areas [4]. There are various poor prognostic factors like the progression of sepsis, multiple abscesses, polymicrobial infection, antibiotic-resistant pathogens, age more than 70 years, abscess associated with neoplasia, and immunosuppression [5,6]

The presentation of an LA is often nonspecific, including fever, abdominal pain, and vomiting, creating a diagnostic quandary; thus, the diagnosis is established based on clinical features and imaging, including an ultrasound or CT scan of the abdomen. Imaging modalities are useful for determining the location and size of the lesion, and image-guided aspiration is critical for determining the type of microbial responsible and its medical treatment [7].

Most abscesses are polymicrobial, with the most commonly implicated microbial for PLA being *Escherichia coli (E.coli)*, *Klebsiella*, *Enterococcus*, *Bacteroides*, and* Staphylococcus* [8]. Most of the time, *E. coli *is responsible in Western countries, but *Klebsiella pneumonia* is responsible in Asian countries [8].

The primary treatment for amoebic LA is medical therapy. However, 15% of LAs are refractory [8]. Further, subsequent bacterial infection may occur in 20% of amoebic LAs [9]. Surgical drainage is the traditional treatment for such patients and those with PLAs, which cause 10-47% morbidity and death [10,11].

Recently, image-guided percutaneous drainage has been increasingly used to treat LAs with a success rate of 70-100% [12-14]. Although the percutaneous placement of an indwelling catheter is the method most widely preferred to drain LAs [15], it is also believed that needle aspiration is simpler, less expensive, and the efficiency is comparable to treating LAs [16].

This study was aimed at understanding the various aspects of LA like demographic profile, clinical presentations, radiological and laboratory findings, treatment modalities, and complications.

## Materials and methods

This prospective, observational study was done in the Department of Surgery, Dr. Sampurnanand Medical College, Jodhpur, Rajasthan, India, from January 2019 to October 2019 after getting ethical approval (SNMC/IEC/2019/56).

Participants

Patients of age more than 18 years and radiologically confirmed cases of LAs were included in this study. After initial management of the patients, and getting written informed consent for inclusion in the study, the patients were assessed by complete blood count (CBC), liver function tests (LFT), renal function tests (RFT), serum electrolytes, blood sugar random, blood coagulation profile, chest x-ray, abdominal sonography, contrast-enhanced computed tomography (CECT) abdomen if required such as in complex or an already ruptured LA. Pus and blood samples were collected and processed as per standard procedures. Patients with immunosuppression, bleeding diatheses, and hydatid cysts were excluded from this study.

Patients were assigned to three groups: (i) Group 1: Medical management alone (in non-aspirable uncomplicated abscess); (ii) Group 2: USG-guided needle aspiration or pigtail percutaneous catheter drainage plus medical management (in unruptured aspirable abscess); (iii) Group 3: Open surgical drainage plus medical management (In ruptured abscesses).

Outcome measures

The response to treatment was assessed by evaluating the time taken in clinical improvement to pain, fever, anorexia, and regression in liver size, time taken to the improvement of LFTs and RFTs, USG evidence of a decrease in size of the abscess cavity, percentage of patients managed conservatively or surgically, duration of hospital stay of patients in days, and percentage of mortality.

Follow-up

Patients were followed up for two months. In the first month, it was once in two weeks, and then at the end of the second month, after discharge, for recurrent attacks or development of complications and to monitor the efficacy of the treatment given.

Statistical analysis

The sample size was estimated as follows: n= z² pq/d², Where n=sample size, p= prevalence of liver abscess 20%, q= free of liver abscess (100%-p% (12%) =88%), d= allowable error (0.10), z= point on normal deviation (1.96) with confidence interval taken as 95%, On calculation, “n” was equal to 40.55, which was rounded off to 41. To compensate for attrition and error, the sample size was increased to 100.

All statistical analyses were performed by using IBM SPSS Statistics for Windows, Version 22.0 (Released 2013; IBM Corp., Armonk, New York, United States). Yates continuity correction test (Chi-square test), Fisher’s exact test, and Fisher-Freeman-Halton test were used for comparison of qualitative data. All data were summarized as mean ± SD for continuous variables, numbers, and percentages for categorical variables. A p < 0.05 was accepted as statistically significant.

## Results

General characteristics

**Figure 1:**
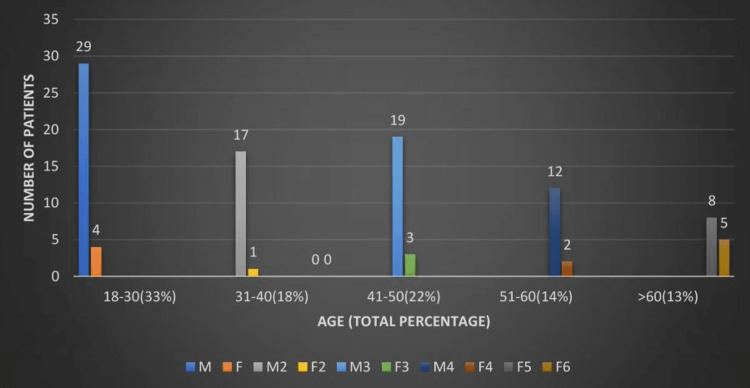
Demographic profile of study population with liver abscess M: male, F: female

A total of 100 patients were included in this study. LA was more common in males (85%) than females (15%) (Figure 1). Peak occurrence was observed in the 18-30 age group (33%) followed by the 41-50 age group (22%).

Fever was the most common presentation observed in 91% of patients (Table 1). Abdominal pain was the second-most common symptom (86%), followed by loss of appetite (43%), jaundice (36%), nausea and vomiting (20%), cough (16%), bowel disturbance (11%), and palpable abdominal lump (4%). Hepatomegaly was the most common sign, followed by tenderness in the right hypochondrium, which was recorded in 62% and 34% of patients, respectively (Table 1). 

**Table 1:** Clinical presentation, physical signs, general physical examination, and the site of pain in liver abscess GPE: general physical examination; RUQ: right upper quadrant; LUQ: left upper quadrant, RLQ: right lower quadrant

Presenting complaints		No. of patients	Percentage
	Fever	91	91.00
	Pain abdomen	86	86.00
	Loss of appetite	43	43.00
	Jaundice	36	36.00
	Nausea &Vomiting	20	20.00
	Cough	16	16.00
	Bowel disturbances	11	11.00
	Abdominal mass	4	4.00
Signs	Hepatomegaly	62	62.00
	Tenderness	34	34.00
	Guarding/rigidity	20	20.00
	Intercostal tenderness	20	20.00
	Ascites	7	7.00
	Splenomegaly	4	4.00
GPE	Icterus	36	36.00
	Pallor	14	14.00
	Dyspnea	2	2.00
	Edema	1	1.00
	Cyanosis	0	0
Site of pain	RUQ	63	63.00
	Epigastrium	23	23.00
	LUQ	5	5.00
	RLQ	4	4.00

**Figure 2:**
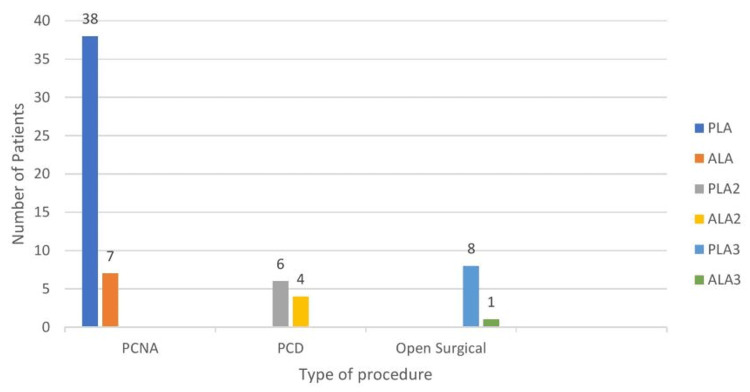
Culture report in different procedures PCNA: percutaneous needle aspiration; PCD: percutaneous pigtail catheter drainage; PLA: pyogenic liver abscess; ALA: amoebic liver abscess

Out of 100, 64 cases were confirmed by culture to have PLA or amoebic LA (Figure 2). Out of these LAs, 52% of patients were having PLAs, where* E. coli *was cultured in 28 (43.75%) and *Klebsiella* in 24 (37.50%) patients. Amoebic LA was present in 12 patients (18.75%).

Out of 100 patients, 33 had a history of alcohol consumption (Table 2). Of those, seven patients were managed medically and 26 were by USG-guided percutaneous needle aspiration, percutaneous catheter drainage, or by open surgery methods. Out of these 26 cases, 24 had PLA and two had amoebic LA. Sixty-seven patients were not consuming alcohol. Of these non-alcoholic patients, 28 had PLA, and 10 had amoebic LA.

**Table 2:** Consumption of alcohol and blood investigations in the study group PCNA: percutaneous needle aspiration; PCD;: percutaneous pigtail catheter drainage; PLA: pyogenic liver abscess; ALA: amoebic liver abscess

Addiction	Medical management	USG-Guided				Open surgical		Total	
		PCNA		PCD					
		PLA	ALA	PLA	ALA	PLA	ALA	N	%
Alcoholic	7	15	2	6	0	3	0	33	33.00
Non-alcoholic	29	23	5	0	4	5	1	67	67.00
Total	36	38	7	6	4	8	1	100	100.00

Twenty-nine patients had a total leucocyte count (TLC) between 4000-10000/mm³ and were mostly managed conservatively by antibiotics alone, and the mean TLC in Group-1 was 11365.27±1051.48/mm³ (Table 3). There were 47 patients with a TLC between 10000-20000/mm³, and 20 patients with TLC between 20001-30000/mm³, while there were only four patients whose TLC was >30000/mm³. 

**Table 3:** Total leucocyte count at admission in the study group PCNA: percutaneous needle aspiration; PCD: percutaneous pigtail catheter drainage; PLA: pyogenic liver abscess: ALA: amoebic liver abscess

TLC (cell/mm^3^)	Medical management	PCNA		PCD		Open surgical procedure	
		PLA	ALA	PLA	ALA	PLA	ALA
4000-10000	27	2	0	0	0	0	0
10001-20000	8	24	5	0	3	6	1
20001-30000	1	10	1	6	1	1	0
>30000	0	2	1	0	0	1	0
Total	36	38	7	6	4	8	1
Mean± SD	11365.27± 1051.48	20242.8± 6484.9		24251.7± 4738.6		17658.8± 8272.9	
p-value	-	0.294		0.126		-	

There were 44 patients with serum bilirubin ≤ 1.1 mg/dl while 33 patients had bilirubin levels between 1.1-2 mg/dl, nine patients had bilirubin levels between 2.1-3 mg/dl, and another nine patients had between 3.1-4mg/dl, only one patient had bilirubin level between 4.1-5 mg/dl, and the bilirubin level was > 5mg/dl in four patients (Table 4). Statistically, the p-value was non-significant.

**Table 4:** Serum bilirubin levels in liver abscess patients PCNA: percutaneous needle aspiration; PCD: percutaneous pigtail catheter drainage; PLA: pyogenic liver abscess; ALA: amoebic liver abscess

S. Bilirubin (mg/dL) Mean (2.08 mgldL)	Medical management	PCNA		PCD		Open surgical procedure	
		PLA	ALA	PLA	ALA	PLA	ALA
<1	17	21	2	0	0	4	0
1.1-2	13	11	3	3	0	3	0
2.1-3	3	3	0	2	1	0	0
3.1-4	2	1	1	1	3	0	1
4.1-5	0	0	1	0	0	0	0
>5	1	2	0	0	0	1	0
Total	36	38	7	6	4	8	1
Mean± SD	1.44± 1.09	2.23± 1.77		2.14± 0.59		2.94± 1.97	
p-value	-	0.433		0.061			

The serum alkaline phosphate (ALP) varied between 65 IU/L and 667 IU/L (Table 5). In patients treated medically, the mean ALP was 213.52±132.2 IU/L, while the ALP in those treated by percutaneous needle aspiration, percutaneous catheter drainage, and by open surgery methods was 209.23±126.44, 313.33±113.56, and 213.62± 89.47, respectively. Out of the 100 patients, nine patients had ALP ≤ 100 IU/L, 49 patients 101-200 IU/L, 15 patients 301-400 IU/L, four patients 3.1-4 IU/L, and four patients had ALP between 401-500 IU/L. Statistically, the p-value is non-significant.

**Table 5:** Serum alkaline phosphate level in liver abscess patients PCNA: percutaneous needle aspiration; PCD: percutaneous pigtail catheter drainage; PLA: pyogenic liver abscess; ALA: amoebic liver abscess

Serum alkaline phosphate (IU/L)	Medical management	PCNA		PCD		Open surgery	
		PLA	ALA	PLA	ALA	PLA	ALA
<100	4	4	0	0	0	1	0
100-200	18	22	4	2	0	3	0
201-300	9	4	1	0	2	2	1
301-400	2	4	2	3	2	2	0
401-500	0	3	0	1	0	0	0
>500	3	1	0	0	0	0	0
Total	36	38	7	6	4	8	1
Mean± SD	213.52± 132.2	209.23± 126.44		313.33± 113.56		213.62± 89.47	
p-value	-	0.544		0.982			

USG detected LA in the right lobe of the liver in 76% of patients (Table 6, Figure 2). In 8% of cases, both lobes were affected and in 16%, only the left lobe had the abscess. Six of nine open surgery patients had a right lobe LA. On USG, 83% of patients had a single LA and 17% had multiple abscesses. Out of 36 cases who were treated conservatively, 30 had single and six had multiple abscesses. Out of the 55 patients treated with USG-guided aspiration or catheter drainage, 46 had single and nine had multiple LAs. Out of nine patients treated with open surgery, seven had single and two had multiple LAs.

**Table 6:** Location, numbers, condition, and volume of liver abscess PCNA: percutaneous needle aspiration; PCD: percutaneous pigtail catheter drainage; S: single; M: multiple

Location of abscess			Medical management	USG-guided					Open surgical			Total	
				PCNA			PCD						
				Pyogenic		Amoebic	Pyogenic	Amoebic	Pyogenic		Amoebic	N	%
	Right lobe	S	24	26		4	5	3	4		1	67	67.00
		M	2	5		1	0	0	1		0	9	9.00
	Left lobe	S	6	4		2	1	1	2		0	16	16.00
		M	0	0		0	0	0	0		0	0	0.00
	Both	S	0	0		0	0	0	0		0	0	0.00
		M	4	3		0	0	0	1		0	8	8.00
	Total		36	38		7	6	4	8		1	100	100.00
Number of abscess	Single		30	30		6	6	4	6		1	83	83.00
	Multiple		6	8	1		0	0	2		0	17	17.00
	Total		36	38	7		6	4	8		1	100	100.00
Condition of abscess	Aspirable		0	37	7		6	4	1	0		55	55.00
	Non-aspirable		36	0	0		0	0	0	0		36	36.00
	Impending rupture		0	1	0		0	0	2	1		4	4.00
	Rupture		0	0	0		0	0	5	0		5	5.00
	Total		36	38	7		6	4	8	1		100	100.00
Volume of abscess (in ml)	Mean ± SD		105.02 ± 72.94	318.88 ± 46.06			552.30 ± 102.04		399.55 ± 142.72				
	P-value			0.285			0.337						

**Figure 3:**
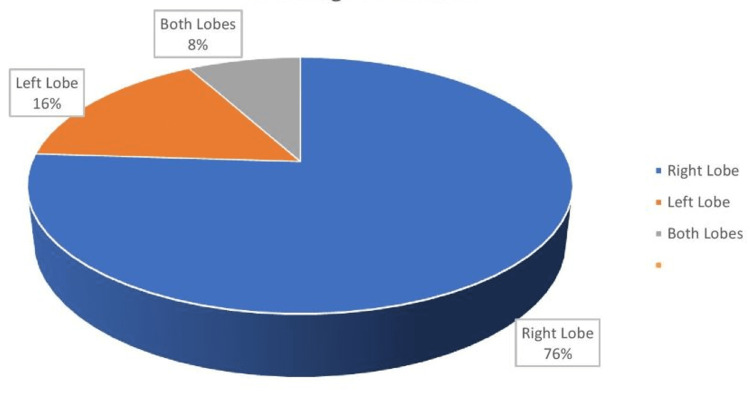
Lobe-wise distribution of liver abscess

In 36% of patients, the LA was non-aspirable, while in 55% it was aspirable. Four percent of patients had LAs that were about to rupture, and 5% had already ruptured LAs when admitted. The mean size of the LA in those who were treated medically was 105.02cc, with a standard deviation of 72.94cc. The mean volume of the LA in the needle aspiration group was 318.88±46.06 cc, and in the pigtail catheter drainage group, it was 552.30±102.04 cc. The mean size of LA in patients treated with the open surgical technique was 399.55±142.72cc.

## Discussion

An LA is the formation of a pus‑filled cavity in the liver parenchyma due to bacterial, fungal, or parasitic infection and is one of the most common tropical diseases of the digestive tract [17,18]. It is broadly classified as PLA or amoebic LA. In our study, LA was more common among patients aged 18-30 (33%), which was younger than in other studies (Figure 1). Mangukiya et al. studied over 400 patients with LAs and found the average age was 35 years [19]. In a 1995 study by Giorgio et al., the mean age of LA cases was 45 years [20]. In our study, the male-to-female ratio was 5.67:1. In groups 1, 2, and 3, the ratio was 2.7:1, 12.75:1, and 8:1, respectively.

Fever was the most common presentation and was seen in 91% of patients, followed by abdominal pain in 86%, loss of appetite in 43%, jaundice in 36%, nausea, vomiting in 20%, and a few other symptoms, including cough expectoration, loose motions, and abdominal mass. These findings were in line with Ochsner et al., who reported that 94% suffered from fever, 92% experienced abdominal pain, and 33% had nausea and vomiting [21]. Greenstein et al. observed that 95% of patients with an LA had a fever, 84% had abdominal pain, 42% had abdominal tenderness, 39% had hepatomegaly, and 24% had jaundice [22].

In 62% of patients, hepatomegaly was the most common sign, followed by jaundice in 36% of cases in our study. Other signs were tenderness in the right hypochondrium, guarding and rigidity, intercoastal tenderness, pallor, splenomegaly, and pedal edema. The majority of male patients (33%) reported alcohol abuse (Table 2). Consuming alcohol renders the liver more susceptible to LA and is linked to larger abscesses, more complications, and higher morbidity [23]. Alcohol impairs liver function and contributes to malnutrition. All of these factors increase the likelihood of developing an LA, especially if they belong to a low socioeconomic condition [24].

The mean serum bilirubin was 2.08 mg/dl and was elevated in 56% of patients, whereas 44% had normal levels (Table 4). The mean levels of bilirubin were 1.44±1.09, 2.23±1.77, 2.14± 0.59, and 2.94±1.97 in medically managed, percutaneous needle aspiration, percutaneous catheter drainage, and by open surgery groups, respectively. Khanna et al. reported jaundice in 46 cases (31.94%) out of 144 amoebic LA cases [25]. Jaundice is caused by intra-hepatic blockage or hepatitis and is frequent in large or numerous abscesses. A porta hepatis abscess causes jaundice due to blockage outside the liver. along with hepatic necrosis with biliary ductal damage could be the most possible hypothesis as the exact pathophysiology is still unclear.

The overall mean ALP was 241.24 IU/L; 213.52±132.2 IU/L in patients with medical management alone, 209.23±124.44 IU/L in percutaneous needle aspiration patients, 313.33±113.56 IU/L in percutaneous pigtail catheter drainage patients, and 213.62±89.47 IU/L in open surgery pattients(Table 5). Khanna et al. reported that ALP was elevated in 76% of cases [25]. Gupta RK found that serum ALP was 24 IU/L after the resolution of the abscess [26]. The mean TLC in those who underwent percutaneous needle aspiration was 20242.8±6484.9/mm³ and for those who were managed by percutaneous catheter drainage was 24251.7±4738.6/mm³. The mean TLC in those who were managed by open surgery was 17658.8±8272.9/mm³ in our study. Ochsner et al. reported a mean TLC of 15000 cells/mm^3^ [21] Sharma et al. found 14800 leucocytes/mm^3^ [27]. According to Lamont et al., a TLC greater than 20,000 cells/mm^3^ implies a subsequent infection in an amoebic LA or PLA [28]. The TLC should drop with clinical, biochemical, and radiological improvement. In our study, 64 of 100 patients with LAs were identified with PLA or amoebic LA, and the aspirate sample was sterile in 26 patients (40.63%), while culture showed growth of *E. coli* in 21 patients (32.81%) and *Klebsiella* in 17 patients (26.56%).

In addition to making the diagnosis, USG was very helpful in demonstrating several other issues, including the number (single or multiple), site (right/left/both lobes/caudate lobe), proximity to porta hepatis with or without intrahepatic biliary radical dilatation, size (with its impact on the biliary tree), and complications like rupture into the peritoneal or pleural cavity, as well as USG-guided aspiration of pus. It also plays a vital role in the follow-up to measure abscess cavity size and see the response of therapy.

We observed that the location of LAs was mostly in the right lobe (76%). Both lobes equally had ruptured LAs. In 83% of patients, the abscess was single, while 17% had multiple abscesses. In a study by Khanna et al., the right and left lobes were affected in 55.5 % and 16.6% of cases, respectively, with multiple abscesses in 27.7% of individuals [25]. Lodhi et al. reported 73% of right lobe amoebic LA, 17% of left lobe, and 10% of both [29].

Out of 100, 83% of patients had normal posteroanterior chest x-rays. The hemidiaphragm was raised in 15% of cases and 11% had pleural effusion. Right-sided effusion was the most common finding in x-rays, which was observed in 81.82% of cases, and 9% had both left-sided as well as bilateral effusion. Patients with normal chest x-ray results could be handled medically; 11% of individuals with pleural effusion and 15% with diaphragm elevation responded to medical therapy alone.

Medical management is sufficient for smaller abscess but the abscess 200 mL or more in size require therapeutic aspiration. Interventional radiology has changed the management of LA over the past three decades. Now, an antibiotic with percutaneous drainage (needle aspiration and pigtail drainage) is used as a preferred management method. Certain criteria for percutaneous drainage include abscess size >5 cm, ongoing pyrexia even after 48 to 72 hours of targeted antibiotic administration, and clinical or imaging features suggesting impending perforation [2]. Many recent studies have used percutaneous drainage in case abscess size >5 cm [20-30,31]. In the study of Kumar et al., 10 cases (40%) were with very large abscesses (>10 cm) and treated with pigtail drainage, and three cases (12%) were with large (5-10 cm) abscesses and treated with needle aspiration, and 12 cases (48%) were treated with only drug therapy alone [32]. Some authors suggest that only antibiotic therapy can be started if the abscess size is less than 3 cm [33,34]. If the abscess ruptures, open surgical drainage combined with medicinal therapy is the optimal treatment. Resolution of the LA was speedier amongst group 3 patients as compared to study group 2. In group 1, the size of the abscess cavity gradually decreased with medical management. In group 2, the size of the abscess cavity gradually decreased by USG-guided needle aspiration or pigtail catheter percutaneous drainage plus medical management. The response to the treatment was almost similar in either variety of abscesses. In group 3, the size of the abscess cavity decreased abruptly in size by open surgical drainage plus medical management. The response to open drainage was marginally better amongst patients with PLAs as compared to those with amoebic LAs.

For those treated medically, the mean volume of the LA was 105.02cc, while the mean volumes were 318.88 cc, 552.30 cc, and 369.55 cc, respectively patients treated with percutaneous needle aspiration, percutaneous drainage, and open surgery. As far as the efficacy is concerned (Table 6), symptoms were completely relieved in 29 patients, symptoms were reduced in intensity in 70 patients, and one patient's symptoms worsened even with management. Sharma et al. noted the death rate to be 0-18% [35], while in our study, one out of 100 patients with LA succumbed to death following intraperitoneal rupture.

## Conclusions

Younger patients between 18 and 30 years of age, especially those with a history of alcohol consumption, are affected more by the pyogenic liver abscess. *E. coli* was found to be the main causative organism, followed by *Klebsiella* in PLAs. Fever and hepatomegaly are the main clinical findings. Patients with small-sized abscesses and near normal leucocyte count and LFTs (ALP, bilirubin) can be well managed by medical treatment. Ruptured abscesses can be effectively treated by open surgical drainage. As needle aspiration is inexpensive and reduces catheter-related complications and long-term hospital stays, and multiple abscesses can be aspirated concurrently through distinct tracts, it's the preferred approach. But in large, resistant LAs, catheter drainage is preferred over needle aspiration; also, aspiration requires repeated needle aspiration in a single patient over a short period, which could be uncomfortable and time-consuming. Therefore, percutaneous pigtail catheter drainage should be recommended as the first-line interventional treatment for LAs.
